# Supplementary material for: High-temperature superconductivity from fine-tuning of Fermi-surface singularities in iron oxypnictides
Source: Sci Rep. 2015 Dec 18;5:18273. doi: 10.1038/srep18273 (PMC4683369; doi:10.1038/srep18273)
Supplement: Supplementary Information [file srep18273-s1.pdf]

High-temperature superconductivity from fine-tuning of Fermi-surface singularities in iron oxypnictides

A. Charnukha[1,2], D. V. Evtushinsky[1], C. E. Matt[3,4], N. Xu[3], M. Shi[3], B. Büchner[1], N. D. Zhigadlo[4], B. Batlogg[4] & S. V. Borisenko[1]

[1]*Leibniz Institute for Solid State and Materials Research, IFW, D-01069 Dresden, Germany,*
[2]*Physics Department, University of California–San Diego, La Jolla, CA 92093, USA,*
[3]*Swiss Light Source, Paul Scherrer Institut, CH-5232 Villigen PSI, Switzerland,*
[4]*Laboratory for Solid State Physics, ETH Zurich, CH-8093 Zurich, Switzerland.*

I. MATERIALS AND METHODS

Angle-resolved photoemission measurements were performed at an angle-resolved photoemission spectroscope ("1^3–ARPES" end-station at BESSY) operating below 900 mK [S 1,2], using synchrotron radiation within the range of photon energies 20-90 eV and various polarizations on *in-situ* cleaved surfaces of high quality single crystals. The overall energy and angular resolution were $\sim$ 4 meV and 0.3°, respectively, for the low-temperature measurements (FS mapping). The FS maps represent the momentum distribution of the intensity integrated within a 15 meV window at the Fermi level. To equalize the intensity over different Brillouin zones the maps were normalised at each **k** point to the total recorded EDC intensity.

High-quality single crystals of superconducting $NdFeAsO_{0.6}F_{0.4}$ with masses of a few micrograms were synthesized by the high-pressure high-temperature cubic anvil technique and were characterized by x-ray diffraction, transport and magnetization measurements [S 3]. The latter revealed a superconducting transition temperature of about 38 K. The width of the superconducting transition was found to be less than 1 K, indicating very high homogeneity of the investigated samples.

II. DETAILED LOW-ENERGY ELECTRONIC STRUCTURE OF $NDFEASO_{0.6}F_{0.4}$ NEAR THE Γ POINT

We have carried out measurements at various energies of the incident X-ray photons to investigate the dependence of the low-energy electronic structure of $NdFeAsO_{0.6}F_{0.4}$ on the out-of-plane momentum $\mathbf{k}_z$. As shown in Fig. S1, neither the surface-related bands, nor, more importantly, the bulk hole band edges show any observable dependence on the photon energy and thus are strongly two-dimensional. Furthermore, the middle column of Fig. S1 clearly demonstrates the presence of two separate bulk bands at binding energies below 56 meV, as argued in the main text and illustrated by dashed lines both in the present figure and in Fig.1 of the main text.

Figure S2 shows a magnified version of the data in Fig.1i of the main text and their derivative to demonstrate the quality of the Lorentz fit of the momentum-distribution curves (black circles in panel **c**) as well as the parabolic fit to the thereby extracted quasiparticle dispersion of the top hole band at the Γ point (white solid line in panel **c**).

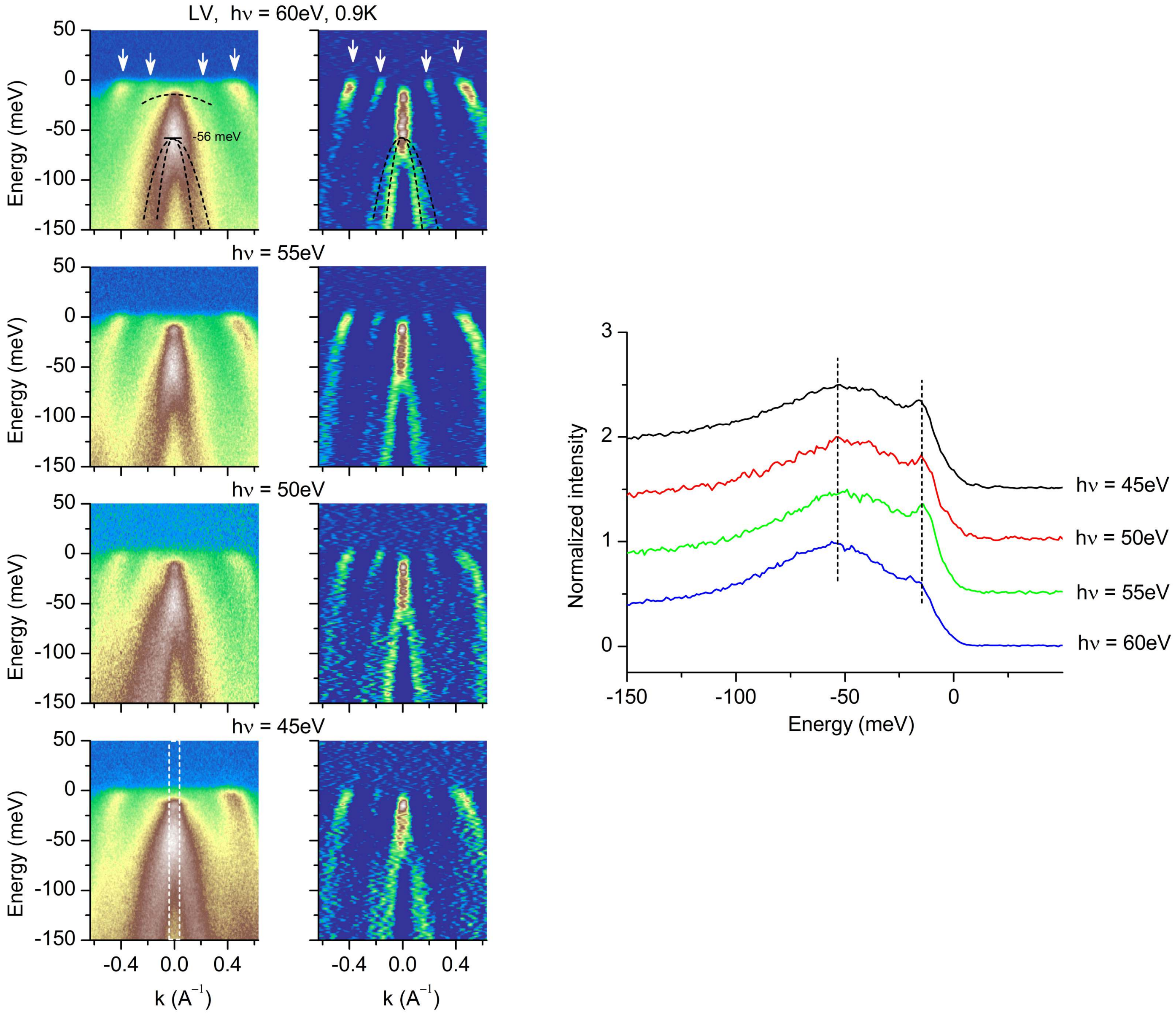

Fig. S1. left column, Energy-momentum cuts taken in the same direction as those in Fig. 1h,i using photons linearly polarized perpendicular to the plane of incidence and with decreasing energy from 60 to 45 eV in steps of 5 eV (top to bottom). White arrows indicate the surface-related bands while the black dashed curves outline the three bulk hole bands, as discussed in the main text. Dashed rectangle in the bottom panel specifies the momentum window used to obtain the integrated energy-distribution curves shown in the right panel. **middle column,** Second derivative of the data shown in the corresponding panels of the left column taken in the direction of the momentum. White arrows and dashed lines are the same as above. **right panel,** Normalized integrated energy-distribution curves obtained by integration in the momentum window shown in the bottom panel of the left column.

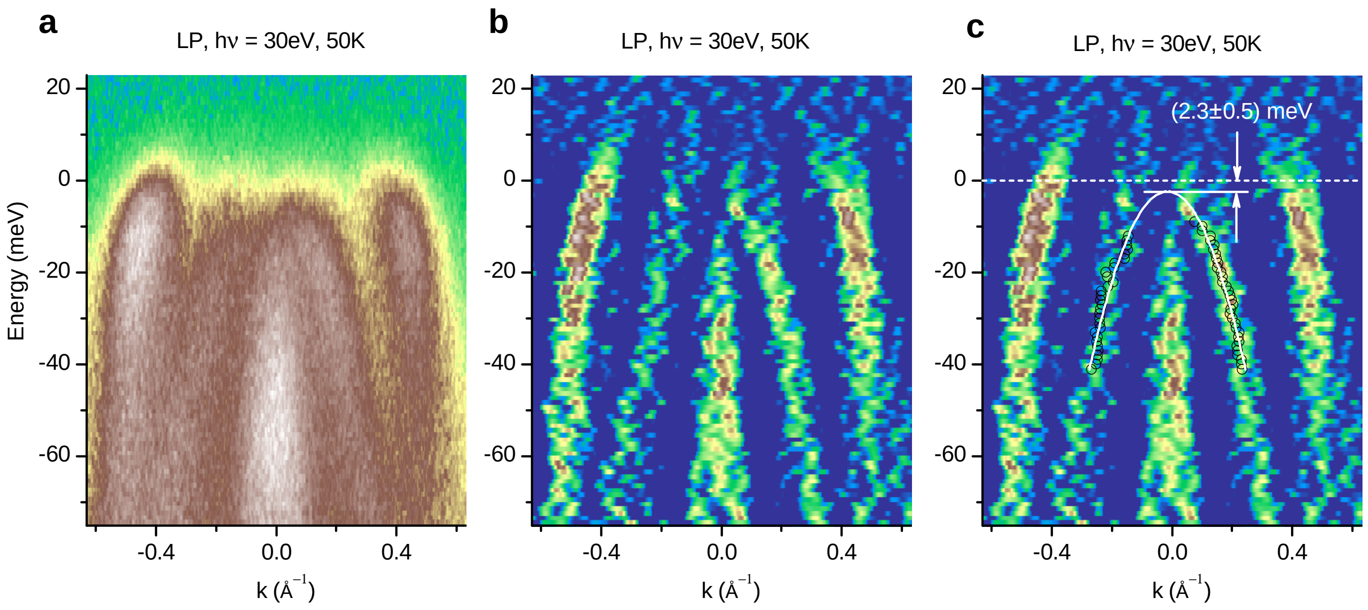

Fig. S2. **a,** Same data as in Fig.1i of the main text. **b,c,** Second derivative of the experimental data in panel **a**. The parabolic fit (white solid line) to the extracted quasiparticle dispersion of the top hole band at the Γ point of the Brillouin zone (black open circles) is the same as in Fig.1i of the main text.

III. TEMPERATURE DEPENDENCE OF THE PHOTOEMISSION INTENSITY NEAR THE Γ POINT

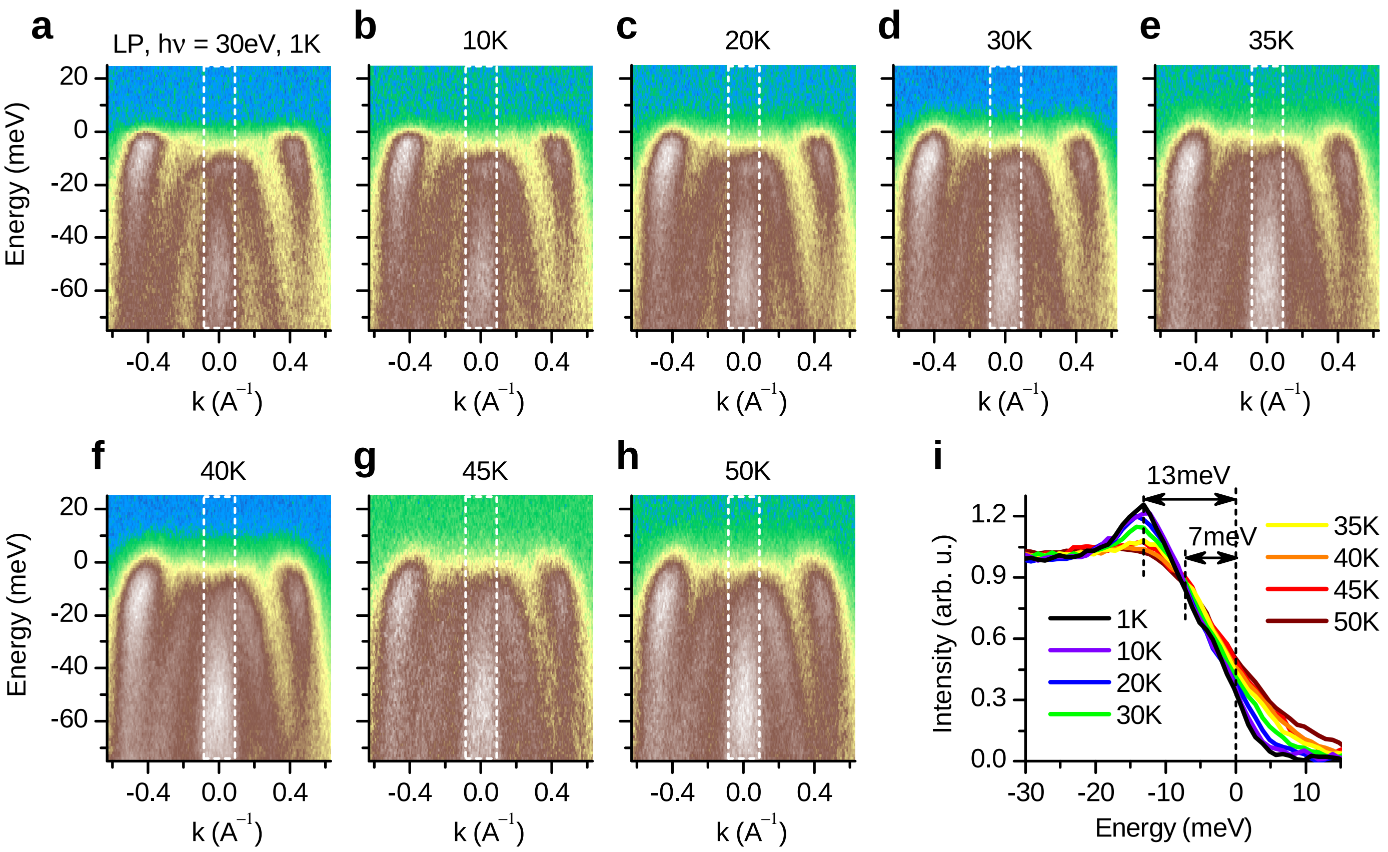

Fig. S3. a–h, Temperature dependence of the photoemission intensity in the energy-momentum cut shown in Fig.2c. Dashed rectangle in all panels specifies the momentum window used to obtain the integrated energy-distribution curves shown in panel **i**. **i,** Energy-distribution curves integrated in the momentum window shown in panels **a**–**h**. Vertical dashed lines indicate the position of (left to right) the superconducting coherence peak, the leading edge of the superconducting component, and the Fermi energy.

References

S1. Borisenko, S. V. “One-cubed” ARPES User Facility at BESSY II. *Synchrotron Radiation News* **25**, 6–11 (2012).

S2. Borisenko, S. V. *et al.* Angle-resolved Photoemission Spectroscopy At Ultra-low Temperatures. *J. Vis. Exp.* **68**, e50129 (2012).

S3. Zhigadlo, N. D. *et al.* High-pressure flux growth, structural, and superconducting properties of *Ln*FeAsO (*Ln* = Pr, Nd, Sm) single crystals. *Phys. Rev. B* **86**, 214509 (2012).
